# STOX1: Key Player in Trophoblast Dysfunction Underlying Early Onset Preeclampsia with Growth Retardation

**DOI:** 10.1155/2011/521826

**Published:** 2010-12-15

**Authors:** Marie van Dijk, Cees B. M. Oudejans

**Affiliations:** Department of Clinical Chemistry, VU University Medical Center, de Boelelaan 1117, 1081 HV Amsterdam, The Netherlands

## Abstract

Currently, only two preeclampsia susceptibility genes (*ACVR2A*, *STOX1*) have been identified within confirmed regions with significant genome-wide linkage, although many genetic screens in multiple populations have been performed. In this paper, we focus on the *STOX1* gene. The epigenetic status of this gene is discussed explaining the maternal transmission of the *STOX1* susceptibility allele observed in preeclamptic families. The known upstream regulation and downstream effector genes of the transcription factor STOX1 are described. Finally, we propose a model in which we combine the cell type-specific and allele-specific effects of STOX1. This includes intrinsic effects (differential CpG island methylation) and extrinsic effects (regulation of effector genes).

## 1. Outline of This Paper

The importance of the *STOX1* preeclampsia susceptibility gene, as discovered in 2005 in Dutch females [1], was initially challenged [2, 3]. However, the latest data including those from independent groups not only confirm the role of *STOX1* in trophoblast dysfunction underlying preeclampsia [4–6] but also indicate a role for its paralog, *STOX2* [7]. Rather than providing a complete overview of all aspects of preeclampsia, we provide a focused overview by summarizing and discussing the latest data on the role of the *STOX1* as a key player in trophoblast dysfunction underlying familial early-onset preeclampsia with growth retardation. We propose a model in which we combine the cell type- and allele-specific (epi)genetic effects of *STOX1* and suggest future directions of research.

## 2. Introduction

Preeclampsia is a pregnancy-associated disease occurring in 5–8% of pregnancies and a major cause of maternal and fetal morbidity and mortality. The disease is characterized by maternal symptoms which may occur from 20 weeks gestation onwards, and which consist of *de novo* hypertension (diastolic blood pressure >90 mm Hg with increment >20 mm Hg from first trimester diastolic blood pressure) and proteinuria (>300 mg per 24 h) as defined by the Royal College of Obstetricians and Gynaecologists [8]. Although the symptoms occur in the mother, it is now well established that the placenta is the driving force; the only cure currently available is delivery of the baby and thus removal of the placenta [9]. 

**Figure 1:**
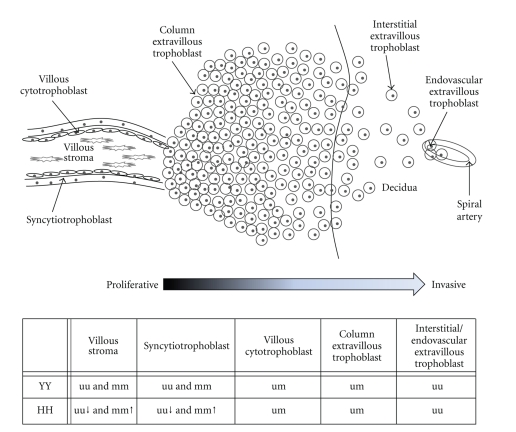
Model of a villus with an extravillous trophoblast column invading the maternal decidua. Below this model, showing different cell types within and originating from a villus, a table shows the *STOX1* methylation pattern observed or hypothesized to be found in the different cell types. YY: placenta homozygous for the Y-allele; HH: placenta homozygous for the H-allele; uu: both alleles are unmethylated; mm: both alleles are methylated; um: imprinting (only one allele is methylated).

In first trimester placenta, insufficient spiral artery remodeling is the fetal pathophysiological origin of preeclampsia [10]. In a normal pregnancy, extravillous trophoblasts from the fetal placenta invade the maternal decidua up to one-third of the myometrium (see also Figure 1). These trophoblasts are thereby transforming the maternal spiral arteries by replacing smooth muscle and elastic tissue with fibrinoid material changing them from low-capacity high-resistance into high-capacity low-resistance vessels. In preeclampsia, this extravillous trophoblast-directed spiral artery remodeling is incomplete leading to a decrease in placental blood flow, which subsequently leads to a response from the mother increasing her blood pressure. This in turn leads to maternal systemic failure giving rise to the maternal symptoms [10].

## 3. Preeclampsia Is a Heterogeneous Disease

It is well established that preeclampsia is a multifactorial disease in which both fetal, that is, placental and maternal, factors are contributing [8]. Different combinations of factors can then lead to differences in disease severity, time of onset, and the occurrence of additional complications, for example, IUGR (Intra-Uterine Growth Restriction). Some of these factors will also run in specific families as there is also a strong genetic component found in the occurrence of preeclampsia [11]. Although already three pathways are known to be involved in preeclampsia, that is, PlGF-sFLT-sENG, TGF*β*-NOS, and COMT-2ME [9, 12], these are all second-order factors that give rise to the maternal symptoms. The first-order causative factors, both placental and maternal, are still largely unknown. One of the important starting points in the identification of these first-order factors is to subcategorize the disease to yield phenotypic homogenous patient populations. The Dutch patients associated with *STOX1* turned out to be phenotypically homogenous as defined by familial early-onset preeclampsia with an abnormal placental development complicated by IUGR [13, 14].

## 4. Preeclampsia Susceptibility Genes Reside in Chromosomal Regions with Demonstrated Genome-Wide Linkage

To identify factors involved in preeclampsia, multiple genetic screens (genome-wide scans, variance-components linkage analysis, and association analysis) have been performed in multiple patient populations (families, case control) [13–20]. This has yielded limited results; only two susceptibility genes have emerged, namely, *ACVR2A* [21] and *STOX1 *[1], both within confirmed regions with significant genome-wide linkage and both involving normal variations (SNPs) in line with the common variant-common disease hypothesis [22]. Both of these genes were originally identified in familial forms of preeclampsia. Additional susceptibility genes and alleles of the same type as found in *ACVR2A* and *STOX1* (common polymorphisms) can also be identified by powerful approaches using genome-wide case-control association analyses. One example, the data are not in the public domain yet, is the ongoing GenPE study by the Wellcome Trust Case Control Consortium (WTCCC2, 2000 cases, 2500 controls) complemented with data from WTCCC3 (2225 cases, 2500 controls) (http://www.wtccc.org.uk). By design, most cases are from nonfamilial forms; the outcome of these case-control studies is therefore bound to be different from, but complementary to, the family-based linkage studies. These studies will predominantly identify maternal susceptibility alleles associated with low to moderate risks and high population frequencies and associated with a low familial component and with the less fatal forms (late-onset without growth retardation). Placental susceptibility alleles with high risks, low population frequencies, and representing the strongly fatal forms (early-onset with growth retardation) with high familial components have been and will be identified by the linkage studies of affected families.

## 5. Familial Preeclampsia Susceptibility Genes

As already mentioned above, only two preeclampsia susceptibility genes have been identified so far, *ACVR2A* [21] and *STOX1* [1]. *ACVR2A*, located on 2q22, was originally identified in an Australian/New Zealand population of preeclamptic pedigrees [21, 23]. Also, in a Norwegian case-control study, SNPs (Single Nucleotide Polymorphisms) within this gene were found to give significant linkage [24]. *ACVR2A* is a receptor for the cell-signaling protein Activin A. Activin A has a major role in promoting decidualization of endometrial stromal cells [25] and in the regulation of trophoblast differentiation and invasion into the decidua [26]. Secondly, multiple studies have found increased Activin A in serum of preeclamptic women [27], making its receptor *ACVR2A* a very interesting protein in the development of preeclampsia. No functional studies have yet been published looking at the significance of the intronic SNPs of *ACVR2A *found in preeclamptic patients [21, 23, 24]. Although intronic SNPs do not appear to be relevant, they should not be discarded as it can be envisaged that these SNPs are within areas controlling *ACVR2A* transcription or are located within novel yet to be identified transcripts.

Our identification of the *STOX1* gene was performed in a Dutch population consisting of affected siblings and their relatives [13]. This identification was preceded by microsatellite marker analysis which revealed a parent-of-origin effect; the alleles shared between affected preeclampsia sisters were always maternal in origin [14]. By sequencing the complete coding region with linkage on chromosome 10q22, the *STOX1 Y153H* common polymorphism was identified, with maternal transmission in three generations [1]. The preeclamptic families with linkage to 10q22 and associated with *STOX1* were a phenotypically homogenous patient cohort suffering from familial severe early-onset preeclampsia complicated by IUGR. This indicates that the disease in these families has a fetal, that is, placental, origin. In this respect, discordant monozygotic twins provide excellent evidence for the placental origin of early-onset preeclampsia; the consistent observation in monozygotic parous twins lacking concordance for preeclampsia implicates that fetal, that is, placental, contributions are essential [28]. Therefore, the discordance seen in monozygous parous twins is in strong favor of genetic but placental origin of preeclampsia. The placental origin of severe early-onset preeclampsia has an important implication in genetic screens; the fetal genotype directs the maternal phenotype [29]. It is therefore essential to realize that research on genetic linkage in familial early-onset preeclampsia should focus on the placental genotype rather than the maternal genotype. For case-control studies, the correction needed and/or allowed is that preeclamptic females born from unaffected and affected mothers should be considered controls and cases, respectively. The importance of the criteria mentioned above can immediately be seen in studies that have tried to confirm the *STOX1 *susceptibility allele in their population [2–4]. Firstly, the preeclamptic women were not clearly defined, the populations consisted of a combination of mild and severe, early- and late-onset preeclampsia, and not always with proof of familial inherited disease. Secondly, as the studies were performed using the maternal genotype, these patients can only be informative if their mother had developed preeclampsia as well. In one study, also performed in a Dutch population, they did take both criteria into account by performing a transmission distortion test (TDT) with correction for grandmaternal origin, and a significant maternal transmission of the *STOX1 Y153H* allele was detected in preeclamptic families when looking across three generations [4]. Recently, another study has been published looking at the *STOX1 Y153H* SNP in the Norwegian population. Again no significant transmission of the H-allele was found when the preeclampsia patients were analyzed in total. But, when looking at recurrent preeclampsia patients, a trend towards significance was detected in H-allele transmission when also corrected for the potential of a parent-of-origin effect [7]. However, these data were obtained in the maternal instead of the placental genotype, thereby diluting the true maternal transmission as not all of these mothers were also born out of a preeclamptic pregnancy. This study, however, also looked at expression of different genes in decidua from preeclampsia, IUGR, preeclampsia complicated by IUGR, and uncomplicated pregnancies; no differences were found in *STOX1 *expression between these groups. Interestingly, a significant difference was observed for *STOX2* gene expression when healthy decidua samples were compared to decidua samples that suffered from preeclampsia complicated by IUGR, where the preeclampsia samples showed a lower expression of *STOX2* compared to controls [7]. Although the analysis was performed in term decidua samples which contain mainly maternal cells, they do consist of approximately 20% placental extravillous trophoblasts. The *STOX2* gene on chromosome 4q35 is located in close proximity to a chromosomal area (4q31-4q32) that has been implicated in preeclampsia in different populations (Finland, Australia, and New Zealand) as well [19, 20, 30]. No function has been allocated to this paralog of *STOX1*, but as it has a high sequence similarity to *STOX1*, some of its functions are potentially comparable. Interestingly, as a comparable function to *STOX1*, which shows a gain-of-function as discussed below, is suggested, and *STOX2* is downregulated in preeclampsia samples, compatible with a loss-of-function, this would indicate that these two genes have similar functions but opposite effects in placental function.

## 6. Epigenetics of STOX1

Although a parent-of-origin effect was already noticed in the first microsatellite analysis [14], as well as maternal transmission of the *STOX1 Y153H* susceptibility allele by sequencing the DNA of multiple generations [1], no (differential) methylation could be detected in the CpG island located within the promoter region of *STOX1* [2, 31], excluding the existence of imprinting of this gene controlled by the CpG island in the promoter. Recently, however, we identified and analyzed another CpG island located in intron 1 of *STOX1 *[32]. Methylation of this intronic CpG region leads to reduced expression in common with the normal association between hypermethylation and downregulation of expression. Secondly, in specific cells, this region is subject to differential and allele-specific methylation. In column extravillous trophoblast samples, the methylated allele is paternal in origin. No allele-specific methylation could be detected in early placenta samples consisting of multiple cell types, but a significant increase in methylation was seen in samples homozygous for the *STOX1 Y153H* preeclampsia susceptibility allele [32]. As column extravillous trophoblasts are formed from villous cytotrophoblasts, we hypothesize that these cells are paternally imprinted as well. In cell types, where methylation is independent of parental origin, the *Y153H* genotype has the potential to direct the level of methylation [32]. How these cell type and allelic differences can be integrated with the function of *STOX1* in the placenta will be discussed in the model proposed below.

## 7. STOX1 Is a Transcription Factor

Three *STOX1* isoforms are known to be expressed with isoform A being the full-length protein. Although also isoforms B and C contain the complete (B) or part (C) of the winged helix DNA-binding domain, they both do not contain a nuclear export signal (NES), only a nuclear localization signal (NLS), keeping them confined to the nucleus and nucleolus, respectively. The general focus therefore is on isoform A as this is the only isoform to contain the regulatory domains needed for its functioning as discussed below. The *STOX1* winged helix DNA-binding domain shows great similarity to the binding domain seen in the family of FOX transcription factors [1]. Its upstream regulation mechanism is also similar as seen in multiple members of the FOX transcription factor family, the PI3K-Akt pathway [6]. When phosphorylated by Akt, the *STOX1* protein is inhibited from entering the nucleus and subsequently degraded by ubiquitination. When phosphorylation does not take place, *STOX1* functions as a transcription factor in the nucleus with binding to DNA. The *Y153H* SNP is located within the winged helix binding domain and affects an amino acid with high molecular effect [1]. By chromatin immunoprecipitation assays, it was established that in the extravillous trophoblast cell line SGHPL-5, the H-allele gives a higher binding affinity as does the Y-allele in one of the *STOX1* downstream effector genes, *CTNNA3* [6]. The *CTNNA3* gene was one of the original candidate effector genes based on its chromosomal location in close proximity to *STOX1*, its function in cell-cell adhesion, and its upregulation found in a preliminary microarray analysis done in *STOX1*-transfected SGHPL-5 cells, an extravillous trophoblast cell line. By studying both SGHPL-5 cells and villous explants, we showed that by binding of *STOX1* to the *CTNNA3* promoter this increased the expression of *α*T-catenin, the protein product of *CTNNA3, *and component of the cell-cell adhesion complex. This in turn leads to reduced trophoblast invasion and maintains proliferation [6]. Furthermore, villous explants homozygous for the *STOX1 *H-allele associated with preeclampsia have a reduced trophoblast outgrowth and an induced responsiveness of *CTNNA3* expression upon *STOX1* expression [6]. In other words, the genotype linked to preeclampsia, that is, *STOX1 Y153H*, has a demonstrated effect on extravillous trophoblast invasion, central in the etiology of preeclampsia. 

The intraintronic gene of *CTNNA3*, *LRRTM3*, is also an effector gene of *STOX1* [33]. Although LRRTM3 is also expressed in placenta, most studies performed on its function are done in brain tissue and cells. This interest comes from the finding that LRRTM3 promotes APP (Amyloid Precursor Protein) processing by BACE1, leading to an increase in Amyloid beta production [34]. Amyloid beta is found to be accumulated in brains of Alzheimer's disease patients. *STOX1* was found to be able to transactivate *LRRTM3* in brain and placenta and showed upregulated expression in brains affected by Alzheimer's disease [33].

A successful genome-wide approach to identify *STOX1* target genes has been described by Rigourd and coworkers [5]. By stable transfection of *STOX1* in the JEG-3 choriocarcinoma cell line as a model for trophoblasts, they identified and confirmed by quantitative RT-PCR five genes upregulated (*PTGDS*, *ALOX5*, *TNFSF10*, *beta-ARRESTIN,* and *TMEM45a*), while four genes were found to be downregulated upon *STOX1* overexpression (*ANXA3*, *ZNF22*, *APOA2,* and *FAM43A*) [5]. Furthermore, by analyzing the promoter regions of genes affected by *STOX1* in the microarray analysis, the most significant promoter binding site being located more than once in each promoter was found to be the FOX transcription factor binding site FKHD. As the *STOX1* DNA-binding domain shows great similarity to FOX transcription factors, promoters containing multiple FKHD sites were chosen for chromatin immunoprecipitation experiments followed by quantitative PCR. Promoter regions of six genes (*COL6A3, S100A4*, *ARRDC3*, *TNFSF10*, *EBI3, *and *PLCB1*) were found to be significantly enriched in cells overexpressing *STOX1* [5]. The microarray data of this study were also compared to published transcriptomic data comparing normal and preeclamptic placentas. The correlation between these studies was found to be highly significant looking at genes affected by *STOX1- *and preeclampsia-modified genes [5]. Interestingly, the microarray data by Rigourd and coworkers has subsequently been used in the study identifying downregulation of *STOX2* in preeclamptic deciduas complicated by IUGR. Their data also found significant correlation between the genes that were up- or downregulated in JEG-3 cells overexpressing *STOX1* and the decidua samples of preeclamptic women [7]. Therefore, although JEG-3 cells were used with *STOX1* overexpression to induce effects, the outcomes are comparable to physiological changes found in preeclampsia samples, justifying the microarray results obtained.

## 8. An Integrated Model of STOX1 in Placental Cells

It has become clear that processes in the early placenta in which STOX1 is involved are cell type dependent and allele dependent. This includes intrinsic effects (differential CpG island methylation) and extrinsic effects (regulation of effector genes) [6, 32]. The cell type-specific effects on expression observed can be combined with the methylation pattern seen in a model depicted in Figure 1. The model shows the methylation status of the different cell types within the early placenta in combination with the *Y153H* allele carried. For simplicity, only the homozygous alleles are shown. It can be seen that, within the villus, stromal cells and syncytiotrophoblasts are not subject to imprinting, but can be either methylated or unmethylated [32]. However, their methylation status is dependent on the *Y153H* allele carried, with the placentas homozygous for the H-allele showing a higher level of methylation than the placentas homozygous for the Y-allele [32]. Although syncytiotrophoblasts in our model are not subject to imprinting, this cannot be ruled out as syncytiotrophoblasts, like extravillous trophoblasts, are originating from villous cytotrophoblasts. The villous cytotrophoblasts are hypothesized to be imprinted within the villus, but as this is only a small population of cells it does not change the overall methylation status observed in early placenta samples. The proliferative column extravillous trophoblasts are proven to be paternally imprinted, but as methylation is less than 50% [32] differentiation of the proliferative extravillous trophoblasts into invasive extravillous trophoblasts is hypothesized to be subject to loss of imprinting. Early placenta column extravillous trophoblasts homozygous for the H-allele show a higher binding affinity to the *CTNNA3 *effector gene than placentas homozygous for the Y-allele [6]. This is followed by a trend towards higher expression of *CTNNA3* mRNA in these cells. This is in agreement with the proposed model as these cells are not subject to differences in methylation level caused by parental independent H-allele directed methylation. This is not the case in the villus part of the early placenta. Although placentas homozygous for the H-allele still will have a higher binding affinity, this is not translated in an upregulated expression of *CTNNA3*; there is even a trend towards downregulated expression in these placentas [6]. This effect can be explained using the model; a vast amount of cells within the villus have a methylation status that is dependent on the allele carried, giving placentas homozygous for the H-allele an overall downregulated expression caused by the higher level of methylation. Furthermore, of interest is the observation that *CTNNA3 *itself is also subject to paternal imprinting in villous cytotrophoblasts [35]. Upon differentiation into proliferative extravillous trophoblasts the expression of *CTNNA3* becomes biallelic and is absent after further differentiation into invasive extravillous trophoblasts.

## 9. Conclusions and Future Directions

This paper has been intended to provide an overview of the data available from successful genetic screens to identify preeclampsia susceptibility genes and subsequent studies on these genes, that is, *ACVR2A *and *STOX1*, in other populations. These two genes are a first step on defining preeclampsia; different genes affect different pathways converging to a final common endophenotype: hypertension with proteinuria. As most data available is on the transcription factor *STOX1*, it has been the main focus of this paper to discuss the epigenetic status of *STOX1*, to explain the maternal transmission seen in preeclamptic families, and its upstream regulation and downstream effector genes in placenta and brain. Finally, we propose an integrated model of the cell type-specific and allele-specific effects on both differential methylation as the expression of effector genes of *STOX1*. 

*STOX1*, however, is only one of the genes that give susceptibility for preeclampsia. It is therefore needed that more genes are identified in other populations to have a complete understanding of the origin of the disease. For new identifications to be successful, it is essential to subcategorize the disease to yield phenotypic homogenous patient populations and to bear in mind that for preeclampsia originating in the early placenta it is the genotype of the children born from these pregnancies that provide information. Secondly, as *STOX1* is a transcription factor, there are many more genes regulated by *STOX1* which need to be identified in a genome-wide manner either by microarrays or by chromatin immunoprecipitation experiments followed by deep sequencing. This will provide a complete overview of *STOX1* function in placenta, brain, and other organs, in both health and disease. Finally, by identifying which other genes are involved in preeclampsia, especially the early-onset form with growth retardation, a complete genotype-phenotype correlation can be envisaged. For this to succeed, functional studies on genes associated with preeclampsia should not only focus on one gene but combine data that is and becomes available. In this respect, it is very interesting to investigate the combined action of the two genes discussed in this paper, *STOX1* and *ACVR2A*, using a physiological *ex vivo* placental explant model which takes into account the different cell types involved [36]. In the end, this will provide a complete functional interaction map of all genes involved in the origin of preeclampsia.
